# Exploring the Influence of Pottery Jar Formula Variables on Flavor Substances Through Feature Ranking and Machine Learning: Case Study of Maotai-Flavored Baijiu

**DOI:** 10.3390/foods14061063

**Published:** 2025-03-20

**Authors:** Haili Yang, Xinjun Hu, Jianpin Tian, Liangliang Xie, Manjiao Chen, Dan Huang

**Affiliations:** 1 School of Mechanical Engineering, Sichuan University of Science and Engineering, Yibin 644000, China; tjp893@126.com (J.T.); xiehnpyll@163.com (L.X.); mj-chen@suse.edu.cn (M.C.); 2 The Liquor Making Biological Technology and Application of Key Laboratory of Sichuan Province, Yibin 644000, China; huangdan3212006@126.com

**Keywords:** pottery jar, metal ions, pore parameters, flavor substances, feature ranking, machine learning

## Abstract

The advantages of pottery jars in the aging process of Baijiu are evident, but the impact of their material composition and pore structure on the flavor of Baijiu has not been widely studied. This study systematically analyzed the effects of six types of pottery jars on metal ions and flavor substances during the storage process of Maotai-flavored Baijiu. It was found that changes in the content of Fe and Zn metals, as well as pore parameters in the jars, significantly affected the content of AL, Mg, K, Na, and Ca ions in Baijiu. Based on three feature ranking methods and three machine learning models, a feature selection method related to flavor substances was established, identifying the key features (i.e., key metal ions) for each flavor group. The final key features of each flavor group can accurately predict the corresponding flavor substance content (Rp^2^ > 0.87). The comprehensive analysis results indicate that the increase in the content of Fe, as well as the increases in P-max and P-min in the pottery jar, collectively promoted the formation of three flavor groups represented by ethyl valerate (G2), ethyl lactate (G7), and ethyl linoleate (G10), with an increase of 3% to 5%. In contrast, the increase in Zn inhibited the formation of the flavor group represented by 2,3-butanediol (G3), with a decrease of 14%. These results further clarify the impact of pottery jar formulations on the changes in flavor substances and provide a more effective method for analyzing the influence mechanism of jars on Baijiu.

## 1. Introduction

Baijiu is a traditional fermented liquor unique to China. With a history that spans thousands of years and having a wide variety of flavors, it is one of the oldest and most consumed distilled spirits in the world [1]. Baijiu comprises 98% ethanol and water, with the remaining 2% consisting of trace amounts of organic acids, esters, and fusel alcohols, as well as trace inorganic compounds [2], which endow the Baijiu with its flavor and determine its style [3]. Following fermentation and distillation, most types of Baijiu are stored in special containers to undergo aging, sometimes for 3–4 years for premium Baijiu. Consequently, storage containers play an important role in the aging process of Baijiu. Pottery jars are the most commonly used storage containers in Baijiu production because they have great air permeability and contain a variety of metal oxides [4]. These metal oxides can leach into the Baijiu, primarily in a free state, which can promote esterification, redox, condensation, and other reactions among the chemical components of Baijiu, thereby influencing the flavor of the product [5]. Before leaving the factory, each batch of Baijiu must undergo testing for contaminants (e.g., heavy metals) and physicochemical parameters (e.g., methanol levels and alcohol content) to ensure compliance with food safety regulations [6,7].

The concentrations of metal ions in Baijiu can significantly influence the formation of organic compounds that ultimately contribute to the flavor of the Baijiu. For example, Baijiu samples with higher metal ion concentrations have been found to have a better taste, indicating a certain correlation between metal ion concentration and the aging process. Therefore, understanding the influence of metal-ion leaching on the change in the chemical composition of Baijiu is key to understanding the mechanism by which pottery jars influence the aging process [8,9]. Huang [10] studied the leaching of metals from pottery jars into strong-flavor Baijiu and the impact of the leached metals on the formation of aroma compounds during storage. They found that the concentrations of Na, K, Ca, Mg, Al, and Fe increased significantly in the Baijiu during storage in the pottery jars. In particular, the Fe^3+^ and Cu^2+^ ions that leached into the strong-flavor Baijiu during storage contributed to the reduction of its organoleptic properties. Xiong [11] stored Baiyunbian Baijiu in different pottery jars for 2 years and found significant differences in the concentrations of Ca^2+^, Fe^3+^, Mg^2+^, and Cu^2+^ ions in the Baijiu. In addition to the leaching of metals from the pottery jars into the Baijiu, Li [12] observed that trace amounts of small molecules within the Baijiu adsorbed onto the surface of the pottery jars. A kinetic model was used to elucidate the adsorption mechanism of the compounds onto pottery powders of different sizes, finding that the adsorption capacity varied depending on the particle size of the pottery powder. While research studies have focused on examining the advantages of using pottery jars to store Baijiu, most have neglected to consider how changes in the composition and pore structure of these jars impact the creation of flavor substances, which necessitates more advanced analytical techniques to explore its intricate connections.

Machine learning (ML) algorithms are highly effective methods for predicting variables by learning data and have been widely applied to large-scale multivariate data analysis [13]. Zhang [14] (2021) used solid-phase, micro-extraction-assisted gas chromatography-mass spectrometry (GC-MS) to conduct non-targeted screening of the volatile components of Baijiu and used partial least squares discriminant analysis (PLS-DA), Spearman’s correlation, and Random Forest (RF) algorithms to analyze the GC-MS data and determine the maturity time of Baijiu. Liu [15] accurately predicted the age of Baijiu samples using deep learning neural networks based on 41 input characteristics. Wei [16] utilized a surface-enhanced Raman scattering (SERS) nanosensor array in combination with a principal component analysis (PCA), support vector machine (SVM), and PLS models to establish a classification model to identify 10 typical flavor substances in Baijiu. Miao [17] combined biological analysis and a BP-ANN prediction model to identify the biomarker for indicating the metabolism under different micro-ecology. Despite these successes, the application of ML models to analyze the complex relationship between the composition and pore structure of the pottery jars and Baijiu flavor has yet to be reported.

This study aimed to investigate how alterations in the composition and pore structure of pottery jars impact the flavor substances, such as organic acids, esters, and alcohols, in Maotai-flavored Baijiu over time. Scanning electron microscopy (SEM), GC-MS, and inductively coupled plasma mass spectrometry (ICP-MS) were used to analyze the composition of the pottery jars and the concentrations of metal ions and flavor substances in the Baijiu. To elucidate the effects of the composition and pore structure of pottery jars on the flavor substance composition in Baijiu, experiments were conducted utilizing six distinct types of pottery jars for the storage of Baijiu. First, partial correlation analysis was conducted to determine the relationship between the parameters of the pottery jars and the metal ions in Baijiu. Then, a clustering analysis of flavor substances was performed to establish flavor groups. Three ML models, combined with three feature ranking methods, were used to create a feature selection method related to flavor substances and to identify the key metal ions that influence the flavor groups. Ultimately, the relationship between the pottery jar and the flavor group was determined indirectly. It is anticipated that the results will further clarify the impact of the pottery jars on the flavor profiles of Baijiu and provide insight into the mechanism by which pottery jars affect Baijiu.

## 2. Materials and Methods

### 2.1. Pottery Jar Samples

The six types of pottery jars were custom-processed at a pottery factory in Sichuan Province, China, with each jar having a volume of approximately 1 m^3^. All pottery jars were fabricated from the same clay material, with the concentrations of Cu, Fe, and Zn ions, as well as the maximum and minimum pore sizes (P-max and P-min), controlled through process adjustments. Notably, during parameter adjustment, the concentrations of Cu, Fe, and Zn ions were exclusively controlled by adding inorganic salts containing these metals. Furthermore, starch was incorporated as a pore-forming agent to alter the pore structure, while all other parameters remained unchanged. The specific parameters are shown in Table 1. The pore structure of each pottery jar was determined by SEM (ZEISS Gemini SEM 300, Carl Zeiss AG, Jena, Germany).

### 2.2. Baijiu Samples

**Figure 1:**
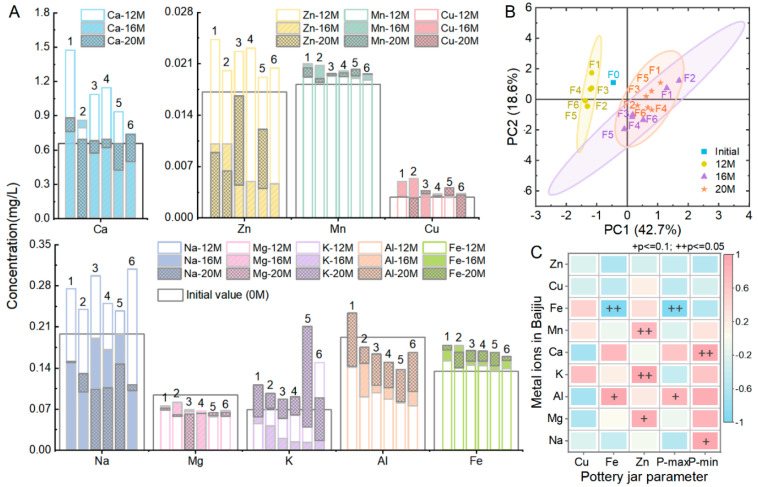
The variations in metal ion concentrations in Baijiu aged for 0, 12, 16, and 20 months in six types of pottery jars. (**A**) Metal ion concentrations in the Baijiu. (**B**) PCA of metal ion concentration in Baijiu. (**C**) Analysis of the partial correlation between pottery jar parameters and metal ions in the Baijiu. + *p* ≤ 0.1 and ++ *p* ≤ 0.05. Red indicates a positive correlation; blue indicates a negative correlation.

The Maotai-flavored Baijiu studied herein was provided by well-known Baijiu enterprises in Sichuan Province, China, and the base liquor used in the experiment was identical. During the storage process, in addition to the initial parameters, the concentrations of the 48 flavor substances (see Section 3.2.2) were determined at 4, 8, 12, 16, and 20 months, while the concentrations of 9 metal ions were only measured at 12, 16, and 20 months (see Figure 1A) due to the relatively slow changes in metal ions in the early stage. Each type of pottery jar was subjected to three sets of parallel experiments. During storage, the pottery jar was sealed with a lid and sealing film and placed in a cellar at 20 °C.

### 2.3. Quantification of Flavor Compounds by GC-MS

The quantitative analysis of 48 flavor substances in each Baijiu sample was carried out by GC-MS (Agilent GC-MS 7890A-5975C, Agilent Technologies, Santa Monica, CA, USA). The DB-WAX capillary chromatography column (60 m × 0.25 mm × 0.25 μm) was used for separation. A total of 5 mL of the Baijiu sample was mixed with 100 μL of a mixed internal standard solution, which included n-pentyl acetate, tertiaryamyl alcohol, and 2-ethylbutyric acid (each at a concentration of 15.10 g/L). The internal standards were obtained from Aladdin Reagent Co., Ltd. (Shanghai, China) and have a purity of at least 98%. Inject 1 µL of the aliquots into the GC-MS instrument for analysis, setting the inlet temperature to 250 °C. The carrier gas used was helium (He), and the flow rate was 1 mL/min in non-split mode. The oven temperature was programmed as: 35 °C (hold 10 min) → 120 °C at 2 °C/min → 200 °C at 5 °C/min → 245 °C at 10 °C/min (hold 40 min). The mass spectrometer was operating in electron ionization (EI) mode (70 eV). The ion source temperature was set to 230 °C, and the interface temperature was set to 250 °C. A solvent delay of 10 min was employed. Mass spectra were acquired in full scan mode across the *m*/*z* 25–550 range.

The identification of compounds was performed by matching the retention indexes (RIs) and mass spectra with reference standards from the NIST17 Library. The standard compounds (Aladdin Reagent, Shanghai, China) were dissolved in a 52% ethanol solution prepared by mixing chromatography ethanol (Sinopharm Chemical Reagent, Shanghai, China) with ultrapure water, followed by serial dilution to prepare six mixed standard solutions. The concentrations of internal standards in the standard solutions were the same as those in the samples. Both the standard solutions and the samples underwent the same extraction and testing procedures. The standard curves were prepared by plotting the ratio of the standard compounds and the internal standard against their concentration ratio and quantified [18].

### 2.4. Quantification of Metals by ICP-MS

The concentrations of nine elements—Ca, Na, Mg, Al, K, Fe, Mn, Cu, Zn—in each Baijiu sample were detected using an X-Series 2 ICP-MS (Thermo Fisher, Waltham, MA, USA) after being aged for 12, 16, and 20 months. The reagents used were 9 single-element standard solutions of Ca, Na, Mg, Al, K, Fe, Mn, Cu, and Zn with a concentration of 1000 mg/L, as well as Rh and Tl single-element standard solutions (Guobiao Testing & Certification, Beijing, China). The Thermo Fisher standard tune solution (Tune A) was utilized. 65% volume fraction nitric acid (Sigma-Aldrich, St. Louis, MO, USA). All experimental water was ultrapure water. First, each Baijiu sample was pretreated by microwave digestion (MARS6, CEM Corporation, Matthews, NC, USA). To do this, a 10 mL sample of Baijiu was placed in the microwave digestion tank, the solutions were concentrated to 4 mL in the acid extractor at 65 °C, and then add 6 mL of nitric acid. Cover the flask and let it stand for 1 h. Subsequently, the tank covered was tightened, and digestion was allowed to proceed according to the standard operation of the microwave digestion instrument. After cooling, the tank cover was slowly opened to release any pressure, and the inner cover was rinsed with a small amount of water. Then, the digestion tank was placed in an ultrasonic water bath to degasify it for 5 min. The volume of each solution was adjusted to 25 mL with water, and the resulting solutions were mixed well and set aside. A blank test was conducted simultaneously. The instrument parameters were optimized for the tuning solution. Internal standards of Rh and Tl (5 µg/L) were used to quantitatively analyze the mixed standard solutions of different elements in CCT mode. Among them, Na, Mg, Al, K, Ca, and Fe were combined together as a mixed standard solution, with series concentrations of 0 μg/L, 20 μg/L, 50 μg/L, 100 μg/L, 200 μg/L, 300 μg/L, 400 μg/L, and 500 μg/L. Mn, Cu, and Zn were grouped together as another mixed standard solution, with concentrations of 0 μg/L, 0.1 μg/L, 0.5 μg/L, 1 μg/L, 4 μg/L, 7 μg/L, and 10 μg/L.

### 2.5. Statistical Analysis and Model Construction

Origin version 2021 (University of Ljubljana, Republic of Slovenia) was used for PCA, partial correlation analysis, correlation analysis, cluster analysis (CA), and data visualization. The Gephi software (version 0.9.2) was used to draw a correlation network diagram using correlation analysis results. The ML model was constructed using R version 4.3.0 (R Statistical Calculation Foundation, Vienna, Austria) and was used to screen key metal ions that affect flavor groups.

Selection of the key metal ions affecting flavor substances and feature evaluation was achieved through a combination of RF, eXtreme Gradient Boosting (XGBoost), and Adaptive Boosting (AdaBoost) algorithms with three feature ranking algorithms—Relief F, F-test of equality of variances (F-test), and Boruta. Specifically, feature ranking was first performed using the three ranking methods, from which corresponding ranked subsets were established (for example, subset 1 includes the first feature, subset 2 includes the first two features, and so on). These subsets were then evaluated using the three ML models—RF, XGBoost, and AdaBoost—to comprehensively select the key metal ions that most significantly affected the composition of flavor substances.

#### 2.5.1. Establishment of Feature Subsets

The feature ranking of the 9 metal ions investigated in the Maotai-flavored Baijiu and the corresponding flavor substances was conducted using three algorithms: Relief F, F-test, and Boruta. The Relief F algorithm randomly selects a sample R from the training sample set each time and then finds the k nearest neighbor samples (near Hits) from the sample set of the same class as R as well as the k nearest neighbor samples (near Misses) from the sample sets of different classes from R. It then updates the weight of each feature to rank them [19]. The F-test is used to conduct significance tests on the means of grouped samples, ultimately selecting features with correlation *p*-values less than 0.01 or 0.05 [20]. The Boruta algorithm is a feature selection algorithm that utilizes the RF classification method and combines duplication with randomness. Each of the original features in the dataset is duplicated to create a shadow feature by randomly permuting the order of the features. An RF classifier is then trained on the new dataset, which features both the original and shadow features. If the classified deems the original features to be more important (have a higher importance score) than the shadow features, those features are considered relevant and are ranked accordingly [21].

#### 2.5.2. RF

RF is an integrated ML algorithm that improves the accuracy and stability of predictions by constructing multiple decision trees. First, a decision tree is constructed by randomly selecting the data samples and features and repeating these steps to construct multiple decision trees. The prediction results are then combined through voting to obtain the final prediction results [22].

#### 2.5.3. XGBoost

XGBoost is a tree-based learning method that fits previously predicted residuals by successively adding CART trees representing each weak learner during training. Adding a tree is equivalent to learning a new function f(x) to fit the previously predicted residuals. When the number of iterations reaches an upper limit, or the residuals stop decreasing, a strong learner consisting of multiple decision trees is obtained. By learning from multiple learners, the model continuously reduces the difference between predicted and actual values to obtain more accurate predictions [23].

#### 2.5.4. Adaboost

Adaboost is an ensemble model that linearly combines multiple weak regressors through continuous iterations to adjust the weights of each weak regressor in order to improve the performance of the overall regression model. Specifically, the process begins by initializing the training set weights: for a training set D containing N samples, the weight of each sample is initialized to 1/N. Then, based on the current sample weights, a weak regressor is trained, and its error rate on the training set is calculated. Next, the sample weights and the current weak regressor’s weight are updated according to the error rate of the weak regressor on the training set. This process is repeated until the preset maximum number of iterations is reached or the error rate falls below a specified threshold. Finally, the predictions of multiple weak regressors are combined through a weighted sum to obtain a strong regressor [24].

#### 2.5.5. Feature Evaluation

Feature evaluation based on model evaluation results. In this study, three parameters—coefficient of determination (Rp^2^) for the test sets, the root mean square error (RMSEP) for the test sets, and the residual prediction difference (RPD) for the test set—were used to evaluate the performance of the prediction model without involving significance tests [25]. An RPD value of greater than 2.4 is typically considered robust. Furthermore, a model is more accurate when the selected prediction model’s R^2^ is closest to 1, and its RMSE is relatively small [26]. The formulas for calculating these parameters are shown below in Equations (1)–(3):

(1) $$R^{2}=1-\frac{\sum_{i=1}^{n}{\left({\hat{y}}_{i}-y_{i}\right)}^{2}}{\sum_{i=1}^{n}{\left(y_{i}-\bar{y}\right)}^{2}}$$

(2) $$RMSE=\sqrt{\frac{1}{n}\sum_{i=1}^{n}{\left({\hat{y}}_{i}-y_{i}\right)}^{2}}$$

(3) $$RPD=\frac{1}{\sqrt{1-R_{p}^{2}}}$$

where *n* is the number of sample sets, ${\hat{y}}_{i}$ and $y_{i}$ are the predicted and measured contents of flavor substances such as lipids and alcohols of Baijiu samples in the training or test sets, respectively, and $\bar{y}$ is the average of the actual values of all samples in the data set.

## 3. Results and Discussions

### 3.1. Influence of Pottery Jar on Metals in Baijiu

Research has shown that the metal ion concentrations in Baijiu vary depending on the type of pottery in which it is stored [27]. These differences were previously determined to be closely related to the metal content of the clay used in the pottery and the manufacturing techniques [28]. Additionally, the microporous structure of the pottery jar facilitates the exchange of oxygen between the external and interior environments, which participates in oxidation and esterification reactions with the components in the liquor. Simultaneously, the metallic components in the pottery jar slowly dissolve into the Baijiu, facilitating esterification and redox reactions, which enhance the smoothness of the Baijiu and create a unique aging flavor [29].

A previous study found that the porous structure of pottery jars and the Ni, Ti, Cu, and Fe ions contained within serve multiple roles in the storage of Baijiu, including oxidation, adsorption, and catalysis [30]. Additionally, a positive correlation was observed between the concentrations of Al, Fe, Mg, K, and Cu ions present in Baijiu and key aromatic compounds such as dimethoxymethane and lactic acid [18].

The study conducted a storage experiment using six types of pottery jars for the same Baijiu. The initial contents of 9 metal ions in the Baijiu were as follows (Figure 1A): Ca (0.656 mg/L), Zn (0.0171 mg/L), Mn (0.0182 mg/L), Cu (0.00285 mg/L), Na (0.198 mg/L), Mg (0.094 mg/L), K (0.0693 mg/L), Al (0.192 mg/L), and Fe (0.135 mg/L). The clay materials for the jars were the same, but there were differences in the concentrations of certain metal ions and the pore structures (Table 1). As shown in Figure 1B, after being stored in six different pottery jars, the principal components of metal ions in Baijiu become increasingly different over time. Figure 1A shows the changes in the concentrations of nine major metal ions in Baijiu after 12, 16, and 20 months of storage. Specifically, after being stored in jar 1 for 20 months, the concentrations of Ca and Al in the Baijiu were 20% and 33% higher than those in the other jars, reaching 0.874 and 0.231 mg/L, respectively. In contrast, the concentration of Mn and Cu in Baijiu stored in jar 2 fluctuated significantly. After storage in jar 3, the levels of Zn in the Baijiu fluctuated significantly, ultimately reaching 0.166 mg/L, which is more than 1.4 times that of the other jars. In jar 5, the levels of K significantly increased after 20 months of storage, reaching 0.209 mg/L, which is more than twice that of the other jars, and the Cu content was also relatively high. In jars 4 and 6, the Zn content fluctuated significantly; after 20 months of storage, its content dropped to 0. Furthermore, in jar 6, the levels of Na, K, and Al in the stored Baijiu fluctuated most significantly, while the Fe content decreased to 0.156 mg/L after 20 months of storage, corresponding to a reduction of 5–11% compared to the other jars.

To further clarify the correlation between the pottery jar formulations and the composition of metal ions in Baijiu, a partial correlation analysis was conducted on the metal ions in Baijiu after aging in the pottery jars for 20 months, and the parameters of the pottery jar (Cu, Fe, Zn, P-max, P-min); the parameters following aging for 12 and 16 months were used as control variables (Figure 1C). The parameters marked in the figure indicate strong significance (+*p* ≤ 0.1 and ++*p* ≤ 0.05). The Fe levels in the pottery jar, and the P-max were both significantly negatively correlated with the concentration of Fe in the Baijiu, while they are significantly positively correlated with the levels of Al in the Baijiu. The Zn levels in the pottery jar were positively correlated with the concentrations of Mg and K in the Baijiu, while the P-min was significantly positively correlated with the levels of Na and Ca in the Baijiu. It was also notable that the amount of Cu in the pottery jar had a relatively small effect on the concentration of any metal ions in the Baijiu. The analysis results showed that the change in the pottery jar formula significantly affected the content of metal ions in Baijiu.

### 3.2. The Influence of Metal Ions on Flavor Substances in Baijiu

The composition of flavor substances in Baijiu, including acids, lipids, and alcohols, are known to be affected by changes in the concentrations of metal ions in Baijiu resulting from the leaching and/or adsorption of metals onto the pottery jar walls. To further explore these relationships, the impact of changes in metal ions in Baijiu on the flavor substances was studied to explore the impact of the pottery jar formulation parameters on the formation of these flavor substances.

#### 3.2.1. Cluster Analysis (CA)

**Figure 2:**
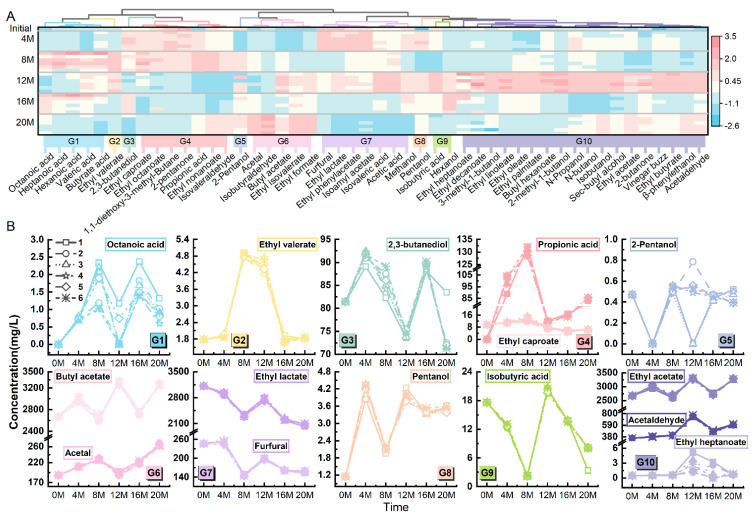
Cluster heatmap and representative parameter changes of the 48 selected flavor substances in Maotai-flavored Baijiu. (**A**) Cluster heatmap, where different colors represent 10 different categories (i.e., 10 flavor groups); (**B**) Changes in the content of 15 representative flavor substances from the 10 flavor groups, where G1, G2… represent group 1 and group 2, and so on.

Each of the Baijiu samples was analyzed using GC-MS. After 12, 16, and 20 months of storage in six types of pottery jars, the content of 48 flavor substances in the Baijiu is shown in Table 2 (Table 2 only shows 3 types of pottery jars, and the storage results of all 6 pottery jars are shown in Table S1). Based on the changes in flavor substances, the K-Means algorithm was used to classify 48 flavor substances into 10 categories (i.e., 10 groups), generating a clustering analysis heatmap of the 48 flavor substances in Maotai-flavored Baijiu (Figure 2A).

To simplify subsequent analysis, one or more flavor substances from the same group can be chosen as representatives, given their similar trends of variation. If the number of flavor substances in the group was less than or equal to 5, one representative compound was chosen; however, if it was greater than 5 but less than 10, two were chosen, and if it was greater than 10, three were chosen. Figure 2B shows the changes in the content of 15 selected representative flavor substances during the 20-month storage period. These compounds include octanoic acid, ethyl valerate, 2,3-butanediol, ethyl caproate, propionic acid, 2-pentanol, acetal, butyl acetate, furfural, ethyl lactate, pentanol, isobutyric acid, ethyl heptanoate, ethyl acetate, and acetaldehyde. Furthermore, flavor compounds within the same group (such as G6, G7, G10) exhibited similar trends in changes despite significant differences in their component concentrations.

#### 3.2.2. Correlation Analysis Based on Key Feature Selection

**Figure 3:**
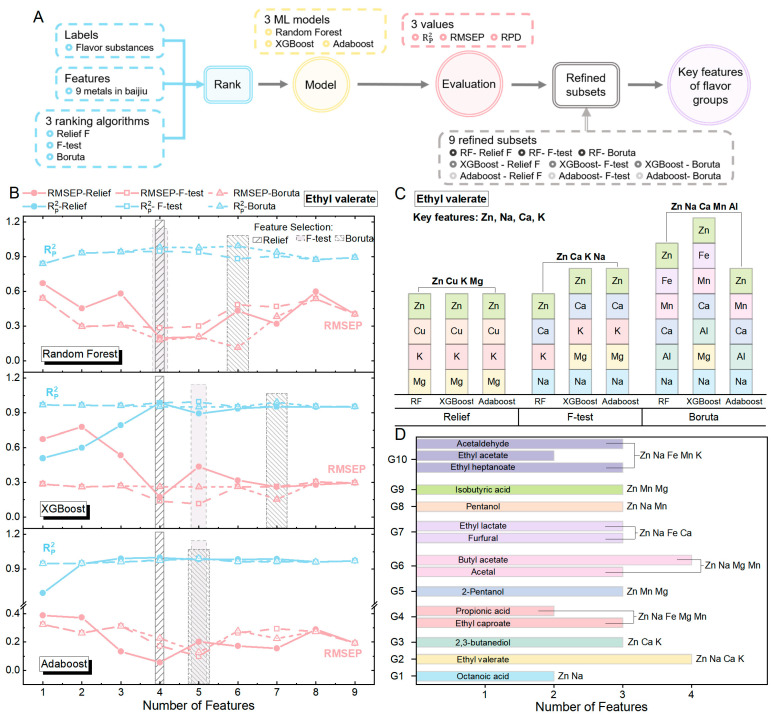
Method and results of key feature selection and evaluation of flavor groups based on machine learning. (**A**) Key feature selection and evaluation process implemented by combining three ML models with three ranking algorithms. (**B**,**C**) Feature selection process using ethyl valerate as an example. (**D**) Key feature selection results of 10 flavor groups.

The study focuses on the Maotai-flavored Baijiu stored in six different types of pottery jars for 12, 16, and 20 months. The partial parameters of flavor substances are shown in Table 2, and the changes in metal ions concentrations are illustrated in Figure 2A. In order to better achieve the selection of key metal ions, 9 types of metal ions were first ranked using 3 feature ranking methods: Relief F, F-test, and Boruta (Figure 3A). The ranking results were then inputted into three ML models—RF, XGBoost, and AdaBoost—to predict the concentrations of specific flavor substances based on the features (i.e., the concentrations of metal ions in the Baijiu) and evaluate selected features. First, for each flavor substance, the features were ranked using an individual ranking algorithm. Then, the sequential forward selection was applied (executing the next subset by adding one feature to the previous subset), where the first subset contains the top 1 feature, the second subset contains the top 2 features, and so on, until the ninth subset contains all 9 features. Consequently, each ranking algorithm can produce nine different input feature subsets. (the results of three ranking algorithms for 15 representative flavor substances are shown in Table S2). Each feature subset is evaluated using the Rp^2^ and RMSEP values of 3 ML models, and the subset that achieves the highest Rp^2^ (nearest to 1) and the lowest RMSEP (nearest to 0) is chosen as the refined subset. Essentially, each ranking algorithm generates three refined subsets, and their common features are identified as a set of key features. Consequently, the three ranking algorithms can generate three distinct sets of key features, and those features that are present in two or more of these sets are designated as the final key features.

Figure 3B shows the Rp^2^ and RMSEP values of the three ML models using the feature subset to predict ethyl valerate (G2). The AdaBoost model more accurately predicted the concentration of ethyl valerate than the RF and XGBoost models. The Rp^2^ value shows a trend of first increasing and then decreasing, while RMSEP shows the opposite trend, which is consistent with the fundamental theory of feature selection. Overall, prediction models built with too few features can lead to a decrease in prediction accuracy, while too many features can introduce noise that interferes with the prediction. Specifically, the feature subsets obtained by the Relief F ranking algorithm were input into the RF, XGBoost, and AdaBoost models, respectively. When the feature subset containing four features was used to predict the content of ethyl valerate, the Rp^2^ and RMSEP values of the three models reached their optimum, with Rp^2^ values of 0.978, 0.947, and 0.998, and RMSEP values of 0.199, 0.283, and 0.056, respectively. Therefore, the three refined subsets obtained all contain four features. The common features among them are Zn, Cu, K, and Mg, which are identified as the key features of this group. Similarly, for the feature subset obtained using the F-test and Boruta ranking algorithms, the corresponding refined subset is obtained by comparing the Rp^2^ and RMSEP values of the model using this subset. Ultimately, two sets of key features were identified: Zn, Ca, K, Na and Zn, Na, Ca, Mn, Al. Select the features that appear twice or more from the three sets of key features as the final key features, which are Zn, Na, Ca, and K. These features significantly influence the formation of pentyl acetate, as shown in Figure 3B,C. In addition, when a flavor group contains several representative flavor substances (such as G5, G7, and G10), the results of their screening should be combined. The results of the feature screening for the flavor group are illustrated in Figure 3D.

By inputting the final key features of flavor groups into three ML models, all of which effectively predicted the representative flavor substances in the corresponding group. Moreover, the prediction accuracy for most cases was higher than that obtained using all nine metal ions (Rp^2^ > 0.87), and the missing values correspond to groups with Rp^2^ values less than 0.85 (Table 3). The results of the analysis show that this feature selection method is viable and suggest that the development of flavor substances is significantly influenced by the combined effects of different metal ions.

**Figure 4:**
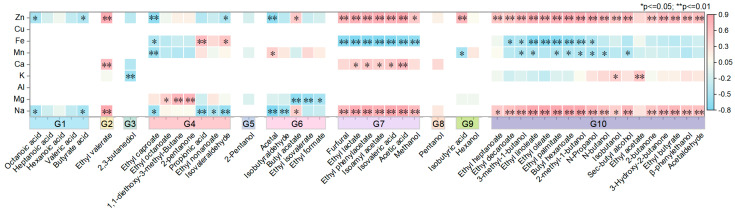
Correlation between key metal ions and flavor substances based on feature selection (* *p* ≤ 0.05 and ** *p* ≤ 0.01; red indicates a positive correlation, blue indicates a negative correlation).

To further understand the relationship between metal ions and flavor substances in Maotai-flavored Baijiu, Pearson’s correlation analysis was conducted between the final key features and flavor substances in the flavor group. Among the 48 flavor substances, 44 showed significant correlations with the selected key metal ions, including 6 acids, 18 lipids, 12 alcohols, 4 aldehydes, 3 ketones, and 1 cycloalkane. For ethyl valerate (G2) was highly positively correlated with the concentrations of Zn, Na, and Ca in the Baijiu, with |r| values ranging from 0.8 to 0.9. In addition, the flavor substances in G7 were highly positively correlated with Zn and Na (0.7 < |r| < 0.9) and highly negatively correlated with Fe (0.7 < |r| < 0.8). Except for furfural and methanol, the flavor substances in G7 were also significantly positively correlated with Ca, while 2-pentanol (G5) and Pentanol (G8) were more weakly correlated with the key metal ions of this flavor group. In general, the results of the correlation analysis between flavor substances and metal ions within the same flavor group are relatively consistent, as illustrated in Figure 4.

### 3.3. The Influence of Pottery Jar on Flavor Substances in Baijiu

**Figure 5:**
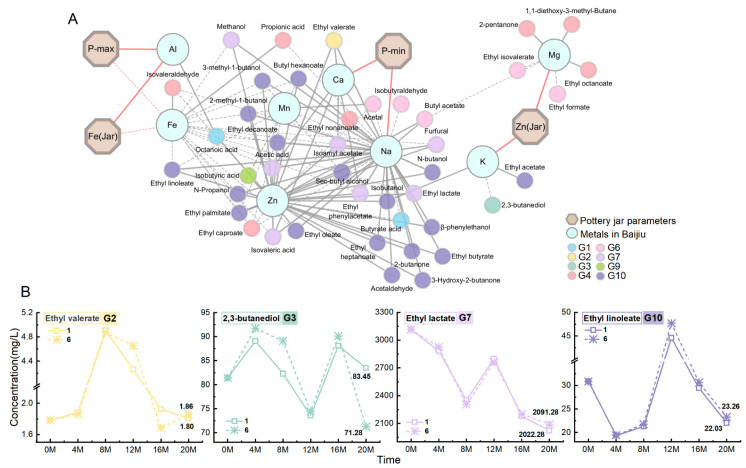
The comprehensive impact of changes in pottery jar parameters on flavor substances. (**A**) Correlation network diagram between pottery parameters, metal ions in Baijiu, and flavor substances; solid lines indicate a positive correlation, while dashed lines indicate negative correlation; the stronger the correlation, the thicker the line. (**B**) Comparison of the content of typical flavor substances in the jars 1 and 6.

By combining the above analysis results (Section 3.2.2) with the partial correlation analysis results of the pottery jar parameters and metal ions (Section 3.1), a correlation network diagram, as shown in Figure 5A, was created. According to the figure, Fe and P-max in the pottery jar were significantly negatively correlated with the content of Fe in the Baijiu, P-min was significantly positively correlated with Na and Ca in the Baijiu, while Zn in the pottery jar was positively correlated with Mg and K in the Baijiu. Furthermore, Na was significantly positively correlated with flavor substances in the G2, G7, and G10 flavor groups, and Ca was significantly positively correlated with flavor substances in the G2 and G7 flavor groups. On the other hand, Fe was significantly negatively correlated with flavor substances in the G7 and G10 flavor groups, while K was significantly negatively correlated with the flavor substances in the G3 flavor group. These results indicate that the increase in Fe content, P-max, and P-min in the pottery jar collectively promotes the formation of flavor substances in G2, G7, and G10 flavor groups, while the increase in Zn content in the pottery jar inhibits the formation of flavor substances in G3 flavor group.

Following the analysis results mentioned above, a further comparison of the content of certain flavor substances in jars 1 and 6 was conducted (Figure 5B). In jar 1, the content of Cu, Fe, and Zn is relatively low, and the P-max is small, while jar 6 has a higher content of Fe and Zn and relatively high P-min and P-max values (Table 1). According to Figure 5B, the concentrations of ethyl valerate (G2), ethyl lactate (G7), and ethyl linoleate (G10) in jar 6 increased by 3% to 5% compared to jar 1. In contrast, the concentration of 2,3-butanediol (G3) significantly decreased by 14%, which aligns with the comprehensive analysis results discussed earlier. Therefore, adjusting the parameters of the pottery jar can promote or inhibit the formation of flavor substances, which is significant for improving the quality of Baijiu, promoting aging and even for the formation of specific flavors.

## 4. Conclusions

Collectively, the analytical and computation results obtained in this study highlight the significant impact of the pore structure and metal composition of pottery jars on the metal ion concentrations and, therefore, the distribution of flavor substances in Baijiu.

From the storage experiment of Maotai-flavored Baijiu using six types of pottery jars (which showed significant differences in Cu, Fe, and Zn metal content as well as pore parameters), it was found that the changes in Fe, Zn, and porosity parameters in the jars significantly affect the composition of metal ions in Baijiu. Through CA, 48 flavor substances were divided into 10 flavor groups. Based on three feature ranking methods and three ML models, a feature selection method related to flavor substances was established, identifying the key features (i.e., key metal ions) for each flavor group. Moreover, the final key features of each flavor group can accurately predict the corresponding flavor substance content (Rp^2^ > 0.87). By combining the correlation network diagram, it was found that the increase in Fe, P-max, and P-min in the jar collectively promoted the formation of flavor substances in G2, G7, and G10 flavor groups, while the increase in Zn in the jar inhibited the formation of flavor substances in G3 flavor group. Moreover, the comparison of representative flavors in jars 1 and 6 also confirmed this result. Specifically, the concentrations of ethyl valerate (G2), ethyl lactate (G7), and ethyl linoleate (G10) in jar 6 rose by 3% to 5% compared to jar 1. On the other hand, the concentration of 2,3-butanediol (G3) saw a notable decrease of 14%.

The research results indicate that adjusting the formula of the pottery jar can promote or inhibit the formation of flavor substances, which is significant for enhancing the aging of Baijiu and even the formation of specific flavors. In addition, this feature selection method that combines feature ranking with machine learning, related to flavor substances, is also applicable to other types of Baijiu. The combination of this method with clustering and correlation analysis helps to clarify the key role of pottery jars in the formation of flavor substances, providing valuable insights for enhancing the formulation of pottery jars.

## Figures and Tables

**Table 1:** Pottery jar parameters.

Number	Cu (mg/L) ^a^	Fe (mg/L) ^a^	Zn (mg/L) ^a^	P-Max (μm) ^a^	P-Min (μm) ^a^
1	0	5.12	0.0077	76.78	5.86
2	0.024	5.28	0.06	84.83	6.42
3	0.088	5.29	0.06	105.16	2.27
4	0.18	5.32	0.15	89.29	2.33
5	0.22	5.19	0.35	74.93	2.42
6	0.11	6.01	0.23	150.48	5.88

^a^ The lowest data detected.

**Table 2:** Content of flavor substances (mg/L) of Maotai-flavored Baijiu in six types of pottery jars (stored for 12, 16, and 20 months).

Flavor Substance	Initial ^a^	12 Months ^a^			16 Months ^a^			20 Months ^a^		
		N_1_	N_3_	N_6_	N_1_	N_3_	N_6_	N_1_	N_3_	N_6_
Acetaldehyde	354.39	770.22	740.07	751.41	499.76	469.83	473.64	609.08	571.53	593.79
Ethyl formate	26.21	70.78	69.4	69.82	58.28	55.21	57.02	99.36	97.12	98.44
Isobutyraldehyde	0	0.04	0.02	0.03	0.04	0.02	0.03	3.63	3.27	3.54
Ethyl acetate	2668.95	3319.56	3284.1	3302.1	2773.44	2712.24	2740.59	3243.26	3229.35	3270.77
Acetal	188.56	198.51	190.77	193.38	222.62	217.2	224.82	261.88	260.49	260.88
2-butanone	5.39	6.7	6.63	6.59	6.03	6.04	6.05	6.11	6.12	6.25
Methanol	105.83	125.87	122.66	121.28	107.98	105.67	102.81	40.57	40.49	42.24
Isovaleraldehyde	42.89	15.78	16.73	18.39	21.54	22.51	24.66	55.92	55.78	56
2-pentanone	2.97	0.02	0.02	0.03	0.02	0.02	0.03	0.02	0.02	0.03
Ethyl butyrate	14.27	19.48	19.05	19.35	16.16	16.05	15.97	16.73	16.69	16.84
Sec-butyl alcohol	55.89	64.68	63.9	64.48	54.87	54.84	54.79	58.36	58.36	58.92
N-Propanol	1052.19	1151.37	1142.55	1148.04	1015.74	1014.39	1014.12	1057.42	1054.77	1065.42
Ethyl isovalerate	2.75	6.61	7.17	6.57	6.82	6.48	6.46	8.37	8.11	8.29
Butyl acetate	1.36	3.89	4.32	3.97	2.74	2.31	2.4	2.96	3.38	2.79
1,1-diethoxy-3-methyl-Butane	16.56	0.03	0.03	0.03	0.02	0.03	0.03	0.02	0.03	0.03
Isobutanol	118.65	135.57	134.36	135.07	117.2	117.14	117.15	128.79	128.49	129.61
Isoamyl acetate	6.28	5.49	5.31	5.59	3.99	4.07	4.05	3.88	4.05	3.9
Ethyl valerate	1.78	4.26	4.73	4.65	1.92	1.73	1.68	1.81	1.85	1.86
2-Pentanol	0.47	0	0	0.54	0.43	0.41	0.45	0.52	0.47	0.4
N-butanol	47.43	54.11	53.51	54.11	45.98	45.91	45.9	49.79	49.72	50.04
2-methyl-1-butanol	56.52	368.06	365.37	366.96	56.52	56.3	56.17	61.78	61.77	62.17
3-methyl-1-butanol	190.53	272.18	270.1	271.57	194.59	193.96	193.85	213.86	213.22	215.23
Ethyl caproate	9.76	7.84	7.82	7.57	6.03	5.11	5.05	6.47	6.42	6.5
Pentanol	1.15	4.24	3.81	3.99	3.33	3.33	3.53	3.57	3.4	3.59
Vinegar buzz	28.85	40.86	40.25	40.74	33.61	33.93	33.67	35.69	34.89	34.45
Ethyl heptanoate	0.45	5.13	3.58	2.76	3.22	0.67	0	0.65	0.63	0.84
Ethyl lactate	3113.46	2798.19	2771.37	2761.83	2178.54	2237.13	2201.58	2022.28	2067.91	2091.28
Hexanol	3.61	5.44	5.24	5.3	6.11	5.69	5.55	3.8	3.82	4.05
Butyl hexanoate	0.87	2.35	2.4	2.15	0.91	0.87	0.9	0.61	0.6	0.54
Ethyl octanoate	5.58	1.11	1.61	2.19	1.78	1.78	1.76	1.41	1.44	1.46
Acetic acid	2375.73	2883.87	2839.95	2827.89	1994.04	1997.91	1963.98	1831.8	1878.21	1898.92
Furfural	246.25	201.48	199.04	198.57	159.76	163.2	160.93	153.34	157.42	159.22
Ethyl nonanoate	1.63	0.02	0.02	0.03	0.02	0.02	0.03	1.23	1.21	1.23
Propionic acid	0	12.24	11.78	11.36	16.96	15.96	15.05	81.03	84.3	85.45
Isobutyric acid	17.57	20.31	20.61	20.5	13.76	13.83	13.51	3.37	8.12	8.05
2,3-butanediol	81.43	73.57	73.79	74.42	88.12	89.78	90.14	83.45	71.52	71.28
Ethyl decanoate	1.14	1.77	1.54	1.41	0.96	1.17	1.32	1.04	0.8	1.26
Butyrate	7.1	17.78	13.25	12.4	19.78	10.64	9.14	8.81	6.09	6.1
Isovaleric acid	21.63	27.44	26.85	26.83	19.77	19.95	19.84	14.53	14.84	15.05
Valeric acid	0.19	2.1	1.56	1.68	3.8	1.51	0.96	0.96	0.42	0.47
Ethyl phenylacetate	5.89	5.73	6.04	5.91	5.01	5.01	4.94	4.58	4.44	4.57
Hexanoic acid	7.69	18	7.55	4.58	64.64	14.21	8.03	6.66	2.92	3.45
β-phenylethanol	12.59	17.04	16.81	16.86	12.52	12.97	12.82	14.61	14.77	14.96
Heptanoic acid	0.03	0.03	0.02	0.02	1.19	0.15	0.19	0.03	0.03	0.02
Octanoic acid	0	1.17	0	0	2.38	1.68	1.4	1.32	0.9	0.91
Ethyl palmitate	43.93	76.07	76.26	76.11	40.62	42.91	41.65	29.55	30.02	30.54
Ethyl oleate	16.42	26.82	26.4	25	16.51	17.16	16.89	10.07	10.26	10.59
Ethyl linoleate	30.83	44.57	50.22	47.68	29.44	31.45	30.6	22.03	22.62	23.27

^a^ The lowest data detected.

**Table 3:** Comparison of results of three ML models.

Flavor Group	Representative Flavor Substance	Feature Number	RF			XGBoost			Adaboost			Final Key Features
			Rp^2^	RMSEP (mg/L)	RPD	Rp^2^	RMSEP (mg/L)	RPD	Rp^2^	RMSEP (mg/L)	RPD	
G1	Octanoic acid	2	0.884	0.326	2.14	0.927	0.219	2.66	0.928	0.181	2.68	Zn Na
		9	0.953	0.164	3.3	0.897	0.159	2.26	0.901	0.391	2.31	
G2	Ethyl valerate	4	0.985	0.169	5.79	0.951	0.311	3.23	0.983	0.187	5.45	Zn Na Ca K
		9	0.893	0.402	2.22	0.952	0.296	3.27	0.971	0.192	4.18	
G3	2,3-butanediol	3	0.863	2.658	1.98	0.983	1.136	5.45	0.975	1.191	4.5	Zn Ca K
		9	0.883	2.501	2.13	0.892	2.664	2.21	0.967	1.498	3.93	
G4	Ethyl caproate	5	0.909	0.407	2.4	0.951	0.254	3.23	0.881	0.219	2.11	Zn Na Fe Mg Mn
		9	0.907	0.298	2.37	0.916	0.27	2.49	0.908	0.305	2.39	
	Propionic acid	5	0.874	0.44	2.06	0.938	0.304	2.88	0.992	0.107	7.92	
		9	0.843	15.19	1.86	0.97	6.435	4.11	0.949	6.409	3.17	
G5	2-Pentanol	4				0.932	0.055	2.76				Zn Mn Mg
		9				0.908	0.382	2.39				
G6	Acetal	4	0.911	9.472	2.42	0.986	3.872	6	0.964	3.993	3.76	Zn Na Mg Mn
		9	0.874	12.49	2.06	0.955	4.984	3.37	0.91	4.789	2.41	
	Butyl acetate	4				0.905	0.279	2.35	0.892	0.29	2.21	
		9				0.794	0.455	1.64	0.93	0.202	2.72	
G7	Furfural	4	0.909	6.387	2.4	0.999	0.444	22.3	0.993	1.676	8.47	Zn Na Fe Ca
		9	0.936	5.454	2.84	0.981	2.726	5.15	0.948	3.816	3.14	
	Ethyl lactate	4				0.999	0.004	22.3	0.998	0.029	15.82	
		9				0.946	75.75	3.08	0.919	16.28	2.54	
G8	Pentanol	3				0.93	0.079	2.72				Zn Na Mn
		9				0.896	0.035	2.25				
G9	Isobutyric acid	3	0.967	1.06	3.93	0.91	2.081	2.41	0.996	0.176	11.19	Zn Mn Mg
		9	0.963	1.08	3.71	0.892	0.952	2.21	0.918	0.32	2.52	
G10	Ethyl heptanoate	5	0.894	0.436	2.23	0.917	0.303	2.51	0.88	0.565	2.11	Zn Na Fe Mn K
		9	0.977	0.167	4.69	0.811	0.506	1.71	0.891	0.433	2.2	
	Ethyl acetate	5	0.896	85.05	2.25				0.989	31.39	6.76	
		9	0.887	91.57	2.17				0.958	56.24	3.49	
	Acetaldehyde	5	0.917	46.98	2.51	0.959	22.44	3.53	0.936	14.26	2.84	
		9	0.829	0.307	1.79	0.885	40.14	2.15	0.959	26.17	3.53	

## Data Availability

The original contributions presented in the study are included in the article/Supplementary Material, further inquiries can be directed to the corresponding author.

## Supplementary Materials

The following supporting information can be downloaded at: https://www.mdpi.com/article/10.3390/foods14061063/s1, Table S1: Content of flavor substances(mg/L) of Maotai-flavored Baijiu in six types of pottery jars (stored for 12, 16 and 20 months); Table S2: Rank scores of different filters.
