# Supplementary material for: Exploring the Influence of Pottery Jar Formula Variables on Flavor Substances Through Feature Ranking and Machine Learning: Case Study of Maotai-Flavored Baijiu
Source: Foods. 2025 Mar 20;14(6):1063. doi: 10.3390/foods14061063 (PMC11941882; doi:10.3390/foods14061063)
Supplement: Supplementary file 1 [file foods-14-01063-s001.zip › foods-3487745-supplementary.pdf]

Table S1. Content of flavor substances(mg/L) of Maotai-flavored Baijiu in six types of pottery jars (stored for 12, 16 and 20 months)

Flavor substances	Initial [a]	12 months [a]					
		N1	N2	N3	N4	N5	N6
Acetaldehyde	354.39	770.22	770.58	740.07	760.29	738.09	751.41
Ethyl formate	26.21	70.78	70.94	69.4	70.33	68.82	69.82
Isobutyraldehyde	0	0	0	0	0	0	0
Ethyl acetate	2668.95	3319.56	3304.62	3284.1	3337.02	3258.9	3302.1
Acetal	188.56	198.51	187.85	190.77	195.78	189.4	193.38
2-butanone	5.39	6.7	6.46	6.63	7.03	6.48	6.59
Methanol	105.83	125.87	116.15	122.66	125.83	121.89	121.28
Isovaleraldehyde	42.89	15.78	20.26	16.73	16.47	16.44	18.39
2-pentanone	2.97	0	0	0	0	0	0
Ethyl butyrate	14.27	19.48	19.05	19.05	19.3	19.06	19.35
Sec-butyl alcohol	55.89	64.68	63.52	63.9	65.06	63.51	64.48
N-Propanol	1052.19	1151.37	1135.35	1142.55	1160.19	1127.97	1148.04
Ethyl isovalerate	2.75	6.61	6.7	7.17	7.06	6.61	6.57
Butyl acetate	1.36	3.89	4	4.32	4.12	3.82	3.97
1,1-diethoxy-3-methyl-Butane	16.56	0.03	0.03	0.03	0.02	0.03	0.03
Isobutanol	118.65	135.57	133.63	134.36	136.38	132.6	135.07
Isoamyl acetate	6.28	5.49	5.35	5.31	5.6	5.27	5.59
Ethyl valerate	1.78	4.26	4.18	4.73	4.43	4.32	4.65
2-Pentanol	0.47	0	0.78	0	0.49	0.56	0.54
N-butanol	47.43	54.11	53.24	53.51	54.4	52.87	54.11
2-methyl-1-butanol	56.52	368.06	362.29	365.37	373.31	360.83	366.96
3-methyl-1-butanol	190.53	272.18	267.41	270.1	274	266.75	271.57
Ethyl caproate	9.76	7.84	6.73	7.82	7.96	7.61	7.57
Pentanol	1.15	4.24	3.96	3.81	3.9	3.77	3.99
Vinegar buzz	28.85	40.86	40.02	40.25	40.95	40.34	40.74
Ethyl heptanoate	0.45	5.13	1.43	3.58	1.14	1.22	2.76
Ethyl lactate	3113.46	2798.19	2736.99	2771.37	2814.66	2715.66	2761.83
Hexanol	3.61	5.44	5.37	5.24	5.12	4.92	5.3
Butyl hexanoate	0.87	2.35	2.22	2.4	2.21	2.19	2.15
Ethyl octanoate	5.58	1.11	2.04	1.61	2.2	0.83	2.19
Acetic acid	2375.73	2883.87	2823.21	2839.95	2876.22	2781.81	2827.89
Furfural	246.25	201.48	197.36	199.04	202.01	195.44	198.57
Ethyl nonanoate	1.63	0.02	0.02	0.03	0.02	0.02	0.03
Propionic acid	0	12.24	11.22	11.78	11.59	11.27	11.36
Isobutyric acid	17.57	20.31	20.35	20.61	20.5	19.66	20.5
2,3-butanediol	81.43	73.57	75.08	73.79	75.02	75.36	74.42
Ethyl decanoate	1.14	1.77	1.34	1.54	2.17	1.58	1.41
Butyrate	7.1	17.78	13.69	13.25	12.83	12.43	12.4
Isovaleric acid	21.63	27.44	26.81	26.85	27.26	26.82	26.83
Valeric acid	0.19	2.1	1.81	1.56	1.81	1.85	1.68
Ethyl phenylacetate	5.89	5.73	6.11	6.04	6.23	5.74	5.91
Hexanoic acid	7.69	18	10.95	7.55	6.18	5.44	4.58
β-phenylethanol	12.59	17.04	16.79	16.81	17.09	16.82	16.86
Heptanoic acid	0.03	0.03	0.02	0.02	1.19	0.15	0.19
Octanoic acid	0	1.17	0	0	2.38	1.68	1.4
Ethyl palmitate	43.93	76.07	76.44	76.26	77.67	74.42	76.11
Ethyl oleate	16.42	26.82	25.98	26.4	26.48	26.18	25
Ethyl linoleate	30.83	44.57	44.07	50.22	48.48	48.7	47.68

Flavor substances		16 months [a]					
		N1	N2	N3	N4	N5	N6
Acetaldehyde		499.76	475.54	469.83	479.97	475.28	473.64
Ethyl formate		58.28	55.95	55.21	56.65	56.21	57.02

Isobutyraldehyde	0	0	0	0	0	0
Ethyl acetate	2773.44	2698.11	2712.24	2737.71	2707.65	2740.59
Acetal	222.62	215.67	217.2	223.96	221.64	224.82
2-butanone	6.03	5.98	6.04	6.04	6.05	6.05
Methanol	107.98	107.04	105.67	104.33	101.75	102.81
Isovaleraldehyde	21.54	20.47	22.51	23.54	24.54	24.66
2-pentanone	0.04	0.03	0.02	0.02	0.03	0.03
Ethyl butyrate	16.16	15.87	16.05	16.11	15.86	15.97
Sec-butyl alcohol	54.87	53.98	54.84	54.89	54.32	54.79
N-Propanol	1015.74	999.27	1014.39	1015.56	1005.75	1014.12
Ethyl isovalerate	6.82	6.35	6.48	6.67	6.43	6.46
Butyl acetate	2.74	2.28	2.31	2.88	2.36	2.4
1,1-diethoxy-3-methyl-Butane	0.02	0.01	0.02	0.01	0.01	0.03
Isobutanol	117.2	115.37	117.14	117.31	116.14	117.15
Isoamyl acetate	3.99	3.97	4.07	4.05	4.08	4.05
Ethyl valerate	1.92	1.81	1.73	1.74	1.67	1.68
2-Pentanol	0.43	0.47	0.41	0.45	0.47	0.45
N-butanol	45.98	45.23	45.91	46.01	45.54	45.9
2-methyl-1-butanol	56.52	55.43	56.3	56.3	55.76	56.17
3-methyl-1-butanol	194.59	190.8	193.96	194.11	192.23	193.85
Ethyl caproate	6.03	4.98	5.11	5.09	4.98	5.05
Pentanol	3.33	3.35	3.33	3.43	3.4	3.53
Vinegar buzz	33.61	33.33	33.93	33.83	33.27	33.67
Ethyl heptanoate	3.22	2.57	0.67	0	0	0
Ethyl lactate	2178.54	2198.52	2237.13	2220.66	2222.64	2201.58
Hexanol	6.11	6.29	5.69	6.28	5.66	5.55
Butyl hexanoate	0.91	0.84	0.87	0.93	0.93	0.9
Ethyl octanoate	1.78	1.74	1.78	1.77	4.62	1.76
Acetic acid	1994.04	1980.72	1997.91	1984.05	1976.31	1963.98
Furfural	159.76	159.98	163.2	162.36	162.5	160.93
Ethyl nonanoate	0.02	0.03	0.02	0.02	0.01	0.03
Propionic acid	16.96	16.29	15.96	15.7	15.5	15.05
Isobutyric acid	13.76	13.69	13.83	13.58	13.65	13.51
2,3-butanediol	88.12	88.55	89.78	88.9	89.84	90.14
Ethyl decanoate	0.96	1.16	1.17	0.98	1.01	1.32
Butyrate	19.78	13.22	10.64	9.83	9.64	9.14
Isovaleric acid	19.77	19.43	19.95	20.17	20.03	19.84
Valeric acid	3.8	2.12	1.51	1.12	1.09	0.96
Ethyl phenylacetate	5.01	4.88	5	4.99	4.79	4.94
Hexanoic acid	64.64	23.3	14.21	7.96	8.63	8.03
β-phenylethanol	12.52	12.72	12.97	12.94	12.97	12.82
Heptanoic acid	1.19	0.39	0.15	0.11	0.17	0.19
Octanoic acid	2.38	1.82	1.68	1.39	1.48	1.4
Ethyl palmitate	40.62	41.21	42.91	42.19	42.8	41.65
Ethyl oleate	16.51	16.96	17.16	17.15	17.32	16.89
Ethyl linoleate	29.44	30.3	31.45	31.16	31.66	30.6
Flavor substances	20 months [a]					
	N1	N2	N3	N4	N5	N6
Acetaldehyde	609.08	607.33	571.53	592.27	595.6	593.79
Ethyl formate	99.36	100.27	97.12	99.36	99.02	98.44
Isobutyraldehyde	3.63	3.53	3.27	3.61	3.43	3.54
Ethyl acetate	3243.26	3266.79	3229.35	3279.02	3288.07	3270.77
Acetal	261.88	269.37	260.48	267.64	264.62	260.88
2-butanone	6.11	6.25	6.12	6.03	6.09	6.25
Methanol	40.57	41.54	40.49	39.33	44.69	42.24
Isovaleraldehyde	55.92	56.05	55.78	57.29	55.02	56

2-pentanone	0.02	0.01	0.02	0.01	0.02	0.03
Ethyl butyrate	16.73	16.8	16.69	16.78	16.96	16.84
Sec-butyl alcohol	58.36	58.7	58.36	58.93	59.17	58.92
N-Propanol	1057.42	1065.27	1054.77	1068.23	1070.46	1065.42
Ethyl isovalerate	8.37	8.73	8.11	8.27	8.31	8.29
Butyl acetate	2.96	3.77	3.38	3.3	3.52	2.79
1,1-diethoxy-3-methyl-Butane	0.02	0.01	0.03	0.02	0.01	0.03
Isobutanol	128.79	129.67	128.49	129.96	130.12	129.61
Isoamyl acetate	3.88	3.96	4.05	3.88	4.04	3.9
Ethyl valerate	1.81	1.82	1.85	1.85	1.84	1.86
2-Pentanol	0.52	0.5	0.47	0.5	0.39	0.4
N-butanol	49.79	50.21	49.72	50.33	50.37	50.04
2-methyl-1-butanol	61.78	62.22	61.77	62.5	139.5	62.17
3-methyl-1-butanol	213.86	215.44	213.22	215.73	139.02	215.23
Ethyl caproate	6.47	6.47	6.42	6.39	6.42	6.5
Pentanol	3.57	3.61	3.4	3.47	3.38	3.59
Vinegar buzz	35.69	34.65	34.89	34.71	36.09	34.45
Ethyl heptanoate	0.65	0.58	0.63	0.73	0.62	0.84
Ethyl lactate	2022.28	2083.15	2067.91	2086.21	2092.53	2091.28
Hexanol	3.8	3.6	3.82	3.61	3.96	4.05
Butyl hexanoate	0.61	0.67	0.6	0.65	0.6	0.54
Ethyl octanoate	1.41	1.4	1.44	1.46	1.42	1.46
Acetic acid	1831.8	1904.97	1878.21	1897.33	1902.94	1898.92
Furfural	153.34	157.56	157.42	158.45	159.26	159.22
Ethyl nonanoate	1.24	1.25	1.21	1.22	1.26	1.23
Propionic acid	81.03	73.73	84.3	85.16	85.48	85.45
Isobutyric acid	3.37	8.24	8.12	8.09	8.07	8.05
2,3-butanediol	83.45	72.49	71.52	71.7	71.96	71.28
Ethyl decanoate	1.04	1.03	0.8	1.18	0.79	1.26
Butyrate	8.81	6.4	6.09	6.06	5.97	6.1
Isovaleric acid	14.53	15.31	14.84	14.97	14.96	15.05
Valeric acid	0.96	0.65	0.42	0.42	0.47	0.47
Ethyl phenylacetate	4.58	4.45	4.44	4.5	4.51	4.57
Hexanoic acid	6.66	4.81	2.92	2.44	2.48	3.44
β-phenylethanol	14.61	14.89	14.77	15.11	14.87	14.96
Heptanoic acid	0.03	0.03	0.03	0.01	0.01	0.02
Octanoic acid	1.32	1.03	0.9	0.59	0.8	0.91
Ethyl palmitate	29.55	30.27	30.02	30.18	30.1	30.54
Ethyl oleate	10.07	10.35	10.26	10.53	10.33	10.59
Ethyl linoleate	22.03	22.62	22.62	22.88	22.27	23.27

[a] The lowest data detected

Table S2. Rank scores of different filters

Flavor substances	Feature ranking methods	Rank scores								
		Na	Mg	Al	K	Ca	Mn	Fe	Cu	Zn
Octanoic acid (G1)	Relief F	0.139	0.095	0.034	-0.003	0.084	0.053	0.047	-0.009	0.140
	F-test	2.237	1.795	1.123	0.440	1.394	1.213	0.147	0.732	2.792
	Boruta	5.523	1.323	1.874	5.286	2.158	5.468	6.876	1.976	7.452
Ethyl valerate (G2)	Relief F	8E-05	0.104	-0.023	0.059	0.040	0.034	-0.018	0.057	0.045
	F-test	2.091	0.949	0.299	1.520	1.542	0.909	0.323	0.233	5.460
	Boruta	2.489	3.99	0.151	5.606	4.883	2.936	-0.461	10.328	2.489
2,3-butanediol (G3)	Relief F	0.107	-0.011	-0.037	-0.021	0.165	0.0002	-0.032	0.042	0.083
	F-test	2.950	0.412	3.38	3.622	5.841	0.950	0.476	0.489	5.420
	Boruta	0.564	6.21	1.544	9.929	7.292	3.373	1.915	0.564	4.046
Ethyl caproate (G4)	Relief F	0.182	-0.016	0.123	0.045	0.037	0.043	0.167	-0.031	0.151
	F-test	5.354	3.377	3.236	1.652	1.192	2.184	4.553	0.265	12.291
	Boruta	6.752	1.872	6.416	4.052	5.012	3.128	1.913	-1.545	8.263
Propionic acid (G4)	Relief F	0.038	0.289	0.075	-0.051	0.029	0.118	0.048	-0.036	0.041
	F-test	0.647	7.685	0.603	0.239	1.122	0.337	0.391	0.762	0.716
	Boruta	8.019	1.1	1.726	6.332	4.316	4.617	2.81	1.818	10.776
2-Pentanol (G5)	Relief F	0.209	0.089	0.072	-0.012	0.060	0.099	0.168	-0.025	0.202
	F-test	9.549	4.450	1.138	2.055	0.719	4.696	2.671	0.249	10.625
	Boruta	2.997	4.503	1.346	0.672	2.601	1.114	2.509	0.998	3.55
Acetal (G6)	Relief F	0.170	0.158	0.074	-0.033	0.049	0.118	0.148	-0.030	0.172
	F-test	5.797	5.262	0.750	1.911	0.547	4.035	2.155	0.234	5.922
	Boruta	8.942	1.636	3.791	8.851	2.047	5.268	8.44	0.324	7.398
Butyl acetate (G6)	Relief F	0.068	0.136	0.024	-0.014	0.034	0.055	0.021	-0.007	0.083
	F-test	1.632	2.670	0.361	0.370	0.850	1.517	0.239	1.100	1.778
	Boruta	7.645	3.637	1.701	2.91	4.866	1.336	0.516	-0.259	6.801
Furfural (G7)	Relief F	0.166	-0.006	0.093	0.067	-0.002	0.043	0.180	-0.023	0.136
	F-test	6.308	4.477	1.491	4.033	1.463	1.949	6.410	0.147	18.069
	Boruta	8.933	1.102	-0.792	0.91	1.006	6.289	6.193	1.078	9.437
Ethyl lactate (G7)	Relief F	0.115	-0.040	-0.037	-0.047	0.157	-0.021	-0.034	-0.055	0.084
	F-test	6.227	0.852	0.077	6.245	1.868	0.332	1.190	0.048	2.209
	Boruta	10.012	0.97	0.425	2.768	1.786	6.849	7.178	0.916	10.013
Pentanol (G8)	Relief F	0.089	-0.012	0.134	0.125	-0.053	0.011	0.171	-0.023	0.045
	F-test	3.196	3.210	1.517	5.480	2.160	0.997	5.400	0.066	20.149
	Boruta	5.951	2.077	-0.195	-0.36	3.35	-2.522	-1.688	-1.304	4.623
Isobutyric acid (G9)	Relief F	0.075	0.121	0.021	0.005	0.060	0.061	0.054	-0.003	0.113
	F-test	5.089	1.788	0.828	1.622	2.150	2.406	0.488	0.409	6.048
	Boruta	9.563	0.572	4.891	7.697	2.459	5.387	6.989	0.408	8.76
Ethyl heptanoate (G10)	Relief F	0.004	0.024	-0.008	0.073	0.017	0.028	-0.002	0.045	0.071
	F-test	4.540	1.206	0.529	3.204	2.773	1.724	0.554	0.125	7.537
	Boruta	8.203	-1.091	0.299	3.241	7.547	2.747	-0.195	-0.174	5.85
Ethyl acetate (G10)	Relief F	0.341	-0.020	0.062	-0.022	0.159	0.027	0.105	-0.051	0.285
	F-test	5.785	2.527	3.064	0.877	1.792	2.959	0.621	0.562	5.775
	Boruta	3.809	4.023	0.081	8.37	7.228	1.075	0.01	1.622	4.119
Acetaldehyde (G10)	Relief F	0.062	0.051	-0.007	-0.005	-0.007	0.105	0.071	0.091	0.077
	F-test	2.624	3.329	1.124	0.409	0.417	5.536	1.645	0.229	8.561
	Boruta	9.682	2.083	5.956	0.128	0.789	3.902	1.753	2.154	9.305
